# Solamargine triggers cellular necrosis selectively in different types of human melanoma cancer cells through extrinsic lysosomal mitochondrial death pathway

**DOI:** 10.1186/s12935-016-0287-4

**Published:** 2016-02-17

**Authors:** Sana S. Al Sinani, Elsadig A. Eltayeb, Brenda L. Coomber, Sirin A. Adham

**Affiliations:** Department of Biology, College of Science, Sultan Qaboos University, P. O. Box 36, 123 Muscat, Oman; Department of Biomedical Sciences, Ontario Veterinary College, University of Guelph, Guelph, ON N1G 2W1 Canada

**Keywords:** Melanoma, Cathepsin B, Solamargine, Cytochrome c, TNFR1

## Abstract

**Background:**

Previous reports showed that the Steroidal Glycoalkaloid Solamargine inhibited proliferation of non-melanoma skin cancer cells. However, Solamargine was not tested systematically on different types of melanoma cells and was not simultaneously tested on normal cells either. In this study we aimed to investigate the effect of Solamargine and the mechanism involved in inhibiting the growth of different types of melanoma cells.

**Methods:**

Solamargine effect was tested on normal cells and on another three melanoma cell lines. Vertical growth phase metastatic and primary melanoma cell lines WM239 and WM115, respectively and the radial growth phase benign melanoma cells WM35 were used. The half inhibitory concentration IC_50_ of Solamargine was determined using Alamarblue assay. The cellular and subcellular changes were assessed using light and Transmission Electron Microscope, respectively. The percentage of cells undergoing apoptosis and necrosis were measured using Flow cytometry. The different protein expression was detected and measured using western blotting. The efficacy of Solamargine was determined by performing the clonogenic assay. The data collected was analyzed statistically on the means of the triplicate of at least three independent repeated experiments using one-way ANOVA test for parametric data and Kruskal–Wallis for non-parametric data. Differences were considered significant when the *P* values were less than 0.05.

**Results:**

Hereby, we demonstrate that Solamargine rapidly, selectively and effectively inhibited the growth of metastatic and primary melanoma cells WM239 and WM115 respectively, with minimum effect on normal and benign WM35 cells. Solamargine caused cellular necrosis to the two malignant melanoma cell lines (WM115, WM239), by rapid induction of lysosomal membrane permeabilization as confirmed by cathepsin B upregulation which triggered the extrinsic mitochondrial death pathway represented by the release of cytochrome c and upregulation of TNFR1. Solamargine disrupted the intrinsic apoptosis pathway as revealed by the down regulation of hILP/XIAP, resulting in caspase-3 cleavage, upregulation of Bcl-xL, and Bcl2, and down regulation of Apaf-1 and Bax in WM115 and WM239 cells only. Solamargine showed high efficacy in vitro particularly against the vertical growth phase melanoma cells.

**Conclusion:**

Our findings suggest that Solamargine is a promising anti-malignant melanoma drug which warrants further attention.

**Electronic supplementary material:**

The online version of this article (doi:10.1186/s12935-016-0287-4) contains supplementary material, which is available to authorized users.

## Background

Metastatic melanoma is the most lethal type of skin cancer, more prevalent within light skinned populations [1]. Although melanoma has lower incidence rate compared with other non-melanoma skin cancers (NMSC) it still counts for 50,000 annual deaths worldwide and it is more common in males compared to females in developed countries [2]. The latest USA cancer statistics report indicated that 43,890 cases were estimated to have melanoma skin cancer and the number of new cases showed an increase in 2014 [3]. Similarly, the incidence of melanoma within Australian susceptible youth is increasing as reported recently [4]. Metastatic melanoma is known for its resistance to chemotherapies notably those with anti-apoptotic action [5–7].

Effective anti-cancer drugs need to selectively kill cancer cells with minimal effect on normal cells. Plant derived compounds such as paclitaxel, originally isolated from the plant *Taxus brevifolia,* are currently used, particularly for the treatment of melanoma and other types of cancer [8]. Increasing evidence shows that Solamargine, a plant derived steroidal glycoalkaloid, also has anti-cancer activity [9, 10]. A previous study proved the efficacy of using a topical cream containing plant isolated Solasodine rhamnosyl glycosides in the treatment of non-melanoma skin cancer [11]. Steroidal glycoalkaloids (SGAs) are naturally occurring nitrogen containing secondary metabolites found in plants of the *Solanaceae* family. The two main compounds are Solasodine and Solamargine, and the main structural difference between them is the chacotriose sugar side chain which is found in Solamargine [12, 13].

Anti-cancer chemotherapies trigger extrinsic and intrinsic apoptotic signals in cancer cells [14]. Solamargine treatment up-regulated the expression of tumor necrosis factor receptors (TNF-R1 and TNF-R2) and the downstream signaling cascades of tumor necrosis factor receptor type 1-Associated Death Domain protein (TRADD) and Fas-Associated Death Domain protein (FADD), along with the activation of the mitochondrial pathway of apoptosis, in human hepatocellular carcinoma, lung cancer cells, breast cancer cells (SK-BR3, MCF-7, HBL-100 and ZR-75-1) [9], human squamous cell carcinoma SCCs [15], and human leukemia (K562) cell lines [16].

However, to our knowledge the effect of Solamargine has not been thoroughly tested on human melanoma skin cancer cells. Therefore, we investigated the effect of Solamargine and the underlying mechanism of action on benign and malignant human melanoma cancer cell lines in vitro. We found that Solamargine selectively inhibited the highly proliferating malignant vertical growth phase (VGP) human melanoma cells lines WM115 and WM239 with minimum effect on the benign radial growth phase (RGP) melanoma cell line WM35, or normal cells. Solamargine induced cellular death in these highly proliferative human melanoma cell lines primarily through the induction of necrosis and rapid rupture of cancer cells via activation of the lysosomal apoptotic pathway. Solamargine disrupted the intrinsic apoptosis pathway as revealed by the down regulation of hILP/XIAP, resulting in caspase-3 cleavage, upregulation of Bcl-xL, and Bcl2, and down regulation of Apaf-1 and Bax in WM115 and WM239 cells only. The clonogenic survival assay proved the efficacy of this drug in vitro particularly against these vertical growth phase melanoma cells. All these findings suggest that the classic caspase-dependent apoptotic pathway is not involved as a mechanism for Solamargine-induced cellular death in susceptible malignant melanoma cells, but rather the extrinsic lysosomal-mitochondrial pathway is responsible. Our results indicate that Solamargine warrants further attention and additional in vivo studies will give a clearer idea of the potential use of this compound as an anti-cancer agent against malignant melanoma.

## Methods

### Drug preparation

The glycoalkaloid Solamargine was obtained from Glycomix Ltd (Whiteknights Road, UK). The drug was dissolved in dimethyl sulfoxide (DMSO) (Sigma-Aldrich, USA) and prepared as stock solution of 1 mM, stored at 4 °C before the experiments and freshly diluted to the final concentrations in culture medium without fetal bovine serum (FBS).

### Cell lines and cell culture

The Human melanoma cell line WM35 was used to represent benign radial growth phase (RGP) primary melanoma [17, 18]. The two cell lines WM115 and WM239 (WM239-A) were used to represent vertical growth phase (VGP) primary melanoma and metastatic melanoma respectively [19]. Cell lines were authenticated by STR analysis, most recently in March 2015 (Genetica DNA Laboratories, Burlington, NC, USA). Primary bovine aortic endothelial cells (BAEC isolated by BLC), rat fibroblast and epithelial cells lines [20] were used in this work as control normal cells. All cells except WM35 were grown in complete culture medium DMEM (Sigma-Aldrich, USA) supplemented with 10 % FBS (Sigma-Aldrich, USA), 1 % (v/v) sodium pyruvate (Sigma-Aldrich, Germany) and 0.5 % (v/v) gentamycin (Gibco, USA). WM35 cells were grown in RPMI media (Gibco, USA), supplemented with 10 % (v/v) heat-inactivated fetal bovine serum (FBS) (Hyclone, UK) 1 % (v/v) sodium pyruvate and 0.5 % (v/v) gentamycin. Cells were grown in a humidified incubator at 37 °C under 5 % CO_2_.

### alamarBlue proliferation assay

1.5–2.0 × 10^4^ cells were seeded in 96-well cell culture plates in 200 μL complete growth medium per well. Following a 24-hour incubation period, the cells were separately treated with serial concentrations of Solamargine and ≤0.1 % DMSO (as control) in 100 μl of FBS free medium at 37 °C for 12 h. Cellular metabolic activity was assessed using alamarBlue assay (TREK Diagnostic System, USA), following the manufacturer’s protocol and the procedure we previously described [21, 22].

### Cell growth curve analysis with and without Solamargine treatment

3 × 10^6^ cells were seeded in 100-mm culture dishes in normal growth medium and incubated at 37 °C in 5 % CO_2_. After 24 h, the cells were treated with Solamargine IC_50_ or ≤0.1 % DMSO (as control) in medium without FBS for 0, 0.5, 1.0, 1.5, 2.0 and 2.5 h. Cells were harvested by trypsinization, stained with trypan blue (Sigma-Aldrich, USA) and live cells were enumerated by standard laboratory method using a hemocytometer. Cell counts were expressed as mean ± standard deviation (SD) of two replicates.

### Mitochondrial membrane potential assay

The JC-1 (tetraethylbenzimidazolylcarbocyanine iodide) mitochondrial membrane potential Assay Kit (abcam, USA) was used to determine the percentage of apoptotic cells (reflected by loss of mitochondrial membrane potential) as a result of solamargine treatment. Fifteen thousand cells of each of cell line were seeded in a 96 well transparent-bottom black plates, allowed to attach overnight and stained with 20 µM JC-1 in 1X dilution buffer for 10 min at 37 °C. Solamargine or the corresponding quantity of DMSO was added to the wells for 2 h, and the plate was read in a fluorescence plate reader (Synergy HTX, Biotek, USA) at Ex475 ± 20 nm/Em530 ± 15 nm. The JC-1 ratio of aggregate/monomer, expressed as percentage of Control was calculated according to the kit instruction manual. This experiment was repeated independently three times and the mean percentage value ± standard deviation were used.

### Clonogenic survival assay

Three million cells were treated with IC_50_ or IC_70_ of Solamargine or ≤0.1 % DMSO (as control) in medium without FBS for 2 h. Following 2 h of incubation, floating cells (conditioned media) were collected, and adherent cells were trypsinized. Conditioned medium and trypsin suspensions were combined and centrifuged for 5 min at 1520×*g*, then re-suspended in fresh growth medium (DMEM with 10 % FBS) and plated in 6 well plates at 10^4^ cells per well. The same steps were performed with control cells. These cells then were incubated for 2 weeks at 37 °C in 5 % CO_2_ chamber, the formed colonies were washed with PBS, stained with crystal violet solution (25 % methanol and 0.5 % crystal violet) and counted manually. This experiment was repeated 3 times by only growing the 10^4^ cells as the best dilution at which the colonies were countable.

### Cell morphology and microscopy

The cells were treated with or without IC_50_ and IC_70_ concentrations of Solamargine or ≤0.1 % DMSO (as control) in medium without FBS. The morphological changes were observed after 2 and 24 h and photographed using inverted phase contrast microscopy (Zeiss, Germany).

### Annexin V measurement of apoptosis

The annexin V-FITC Apoptosis Detection Kit (BioVision, USA) was used to detect and quantify apoptosis by flow cytometry according to the manufacturer’s instructions. In brief, 3 × 10^6^ cells were seeded in 100-mm dishes in normal growth medium. The next day, cells were treated with IC_50_ and IC_70_ of Solamargine, or ≤0.1 % DMSO (as control) in medium without FBS for 2 h. Then the cells which floated in the conditioned media were washed with PBS and collected by centrifugation for 5 min. Cells were then re-suspended at a density of 1–5 × 10^5^ cells in 500 µl of binding buffer and stained simultaneously with FITC-labeled annexin V (5 µl) and propidium iodide (PI) (5 µl). Cells were incubated at room temperature for 15 min in the dark. Annexin V-FITC binding was analyzed by flow cytometry (FACS Aria III, BD Biosciences, USA) using FITC signal detector and propidium iodide (PI) staining by the phycoerythrin emission signal detector. The data were analyzed using BD FACSDiva™ software. A minimum of 10,000 events were acquired per sample.

### Transmission electron microscopy analysis

For electron microscopic observation, 3 × 10^6^ cells were seeded in normal growth medium. The next day, cells were treated with IC_70_ of Solamargine or ≤0.1 % DMSO (as control) in medium without FBS for 2 h. Floating cells in the conditioned media were centrifuged for 5 min at 1520×*g* washed with 1 ml PBS and collected by centrifugation for 5 min. 1 × 10^6^ cells were washed by 200 µl of PBS and centrifuged for 3.5 min at 200×*g*, fixed in 2.5 % glutaraldehyde prepared in 0.1 M sodium cacodylate buffer—pH 7.2–7.4 for 1 h on ice. Cells were rinsed twice with isotonic buffer (0.1 M sodium cacodylate buffer—pH 7.2–7.4) each for 5 min on ice. Samples were then post-fixed in 1 % OsO_4_, dehydrated and embedded in pure EPOXY resin kit (Agar scientific, UK), labeled, and polymerized at 60 °C overnight. Ultra-thin sections (70 nm) were produced and stained with uranyl acetate and lead citrate super saturated in 50 % ethanol. Two Sections from each treatment were then examined using JEOL1230 transmission electron microscope (JEOL, USA).

### Fluorescent nuclear staining

The cells (15,000) were grown on slides in 10 cm well plates, fixed with 4 % paraformaldehyde for 15 min, washed 3 times for 5 min each with PBS and stained with 10 μg/mL propidium iodide (PI) in PBS for 5 min. Slides were then washed in PBS, mounted with fluorescent mounting media (Dako) and observed under fluorescent microscope using the Ex530 nm/Em615 nm filters for PI staining.

### PAGE and western blot

3 × 10^6^ cells were treated with either IC_50_ or IC_70_ of Solamargine or ≤0.1 % DMSO (as control) in medium without FBS for 2 h. The whole cell lysates were prepared by using ice-cold cell lysis buffer (Cell Signaling Technology, USA) mixed with 1 % (v/v) protease inhibitor phenylmethylsulfonylfluoride (PMSF) (Millipore, USA) and 1 % (v/v) protease inhibitor cocktail (Sigma-Aldrich, USA). The cell lysates (100 μg total protein) were loaded in 10 % SDS-PAGE and electrophoretically transferred to Immunobilon-PSQPVDF membrane (EMD Millipore, USA). The membranes were blocked for 30 min in 5 % nonfat milk then incubated overnight at 4 °C with the respective primary antibodies (Additional file 1: Table S1) (diluted 1:1000 in the same blocking solution). Membranes were then washed and incubated for 30 min with the appropriate secondary antibody at dilution of 1:20,000 conjugated with horseradish peroxidase (Cell Signaling Technology, USA). The blots were incubated with enhanced chemiluminescence (ECL) solution (mouse/rabbit) (Roche, Germany) and protein bands visualized by subjecting them to a sensitive X-ray film (Roche, Germany).

### Statistical analysis

All results are presented as mean ± standard deviation (SD). Statistical analysis was performed on the means of the triplicate of at least three independent repeated experiments using one-way ANOVA test for parametric data and Kruskal–Wallis for non-parametric data. Differences were considered significant when the *P* values were less than 0.05.

## Results

### Solamargine had a selective inhibition effect on melanoma cell proliferation

**Fig. 1:**
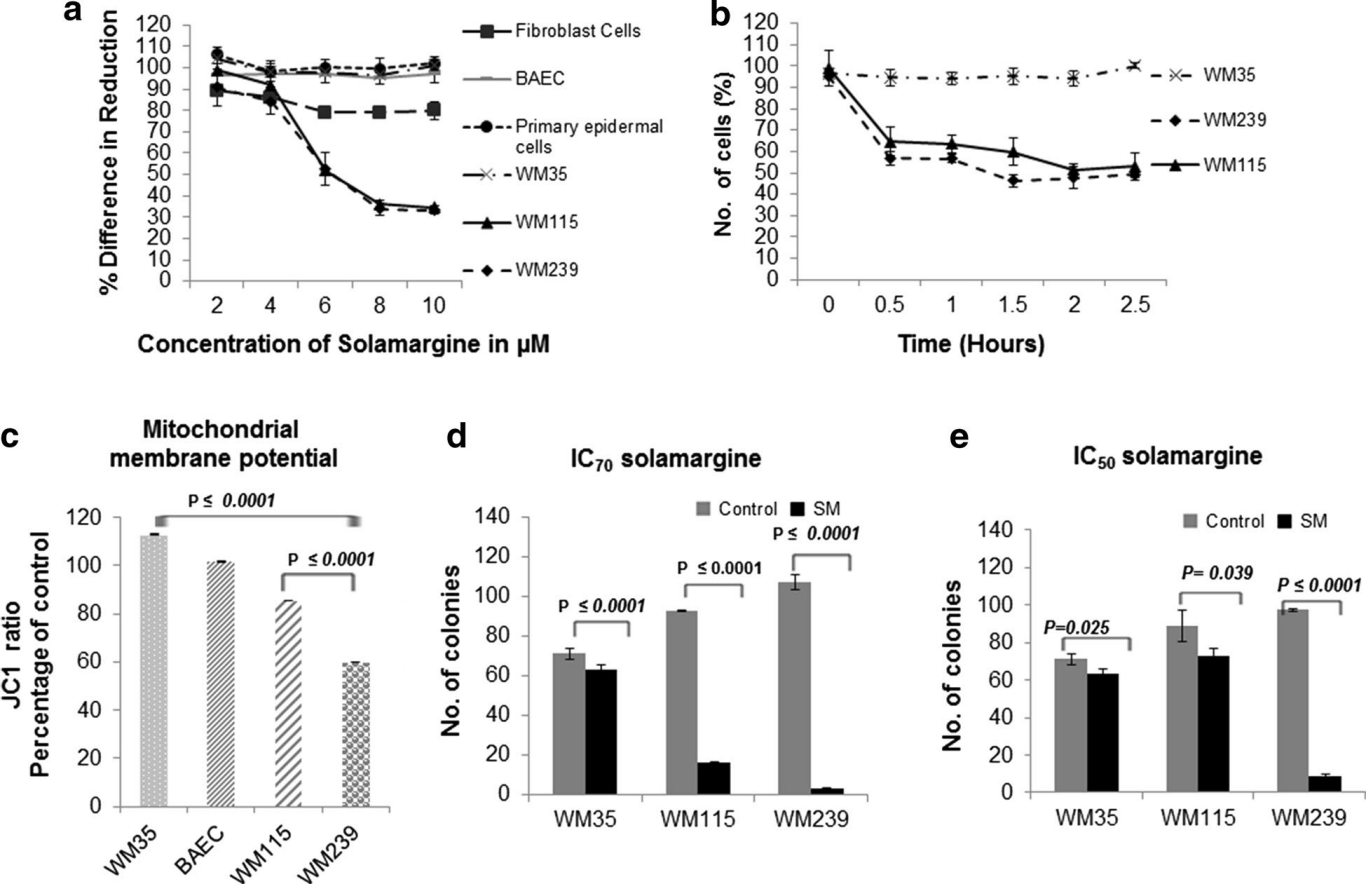
Dosage and time effect of Solamargine on cellular proliferation and viability. **a** Proliferation of WM115 and WM239 as measured by alamarBlue assay was reduced by 50 % upon treatment with 6 µM (IC_50_) of Solamargine, while benign WM35 and the normal cells did not show significant reduction in proliferation at doses as high as 10 µM. The *graph* represents the mean ± S.D (*error bars*) of four independently repeated experiments. **b** Viability of WM115 and WM239 was reduced by 50 % after 30 min of Solamargine IC_50_ administration; however WM35 cell viability was not significantly affected even after 2.5 h of drug administration. **c** Mitochondrial membrane potential as revealed by JC-1 aggregate signal was significantly reduced in WM239 cells and to a lesser extent in WM115 cells, when compared to control untreated cells. There was no significant reduction in mitochondrial membrane potential in either WM35 benign melanoma cells or normal BAEC cells. **d** and **e** The IC_50_ (6 µM) and IC_70_ (10 µM) of Solamargine treatment significantly reduced colony formation ability of all the melanoma cell lines in the study; the most profound decrease was noticed in WM239, N = 4, p < 0.0001

The drug concentrations that induced 50 % (IC_50_) and 70 % (IC_70_) of melanoma cell death were calculated after performing alamarBlue proliferation assay (in four independent experiments) as shown in Fig. 1a. IC_50_ and IC_70_ were found to be 6 and 8 µM for VGP primary melanoma WM115 and metastatic melanoma WM239 respectively. Increasing the drug concentration from 8 to 10 µM did not change the percentage of reduced alamarBlue in WM239 cells. However, the RGP primary melanoma cell line WM35, fibroblast cells, primary epidermal cells and BAEC did not show any significant reduction in their proliferation even at 10 µM (Fig. 1a). 20 µM was also used to check the proliferation of the WM35 cells and there was no effect cell proliferated (data not shown), therefore, we used the upper limit of 10 µM to represent the IC_70_.

### Growth curve and time course effect of Solamargine on melanoma cellular death

In order to assess the effective time of Solamargine (IC_50_) cytotoxicity on melanoma cells, cellular death was assessed by counting the number of remaining adherent cells every 30 min after the administration of the drug for a period of 2.5 h. Solamargine IC_50_ induced loss of WM115 and WM239 cells within the first 2 h of treatment; however Solamargine did not result in any detectable cellular death (drop in cellular count) of WM35 cells within the study period and even after 24 h of drug administration (Fig. 1b).

### Solamargine profoundly reduced the mitochondrial membrane potential in the metastatic cell line WM239

Quantification of changes in mitochondrial membrane potential showed that the metastatic VGP melanoma cells WM239 were the most affected by Solamargine treatment (Fig. 1c). The JC-1 aggregate signal was reduced significantly (*P* = 0.0019) by 40 % when compared to the control cells or to primary lesion WM115 cells, which showed a reduction by 14.6 % compared to control (*P* ≥ 0.001). Normal BAEC and the benign WM35 cells did not show any significant reduction in mitochondrial membrane potential with Solamargine treatment (Fig. 1c; *P* > 0.05*)*.

### Solamargine treatment resulted in a differential reduction in the ability of melanoma cells to form colonies

Clonogenic survival assay was performed to assess the ability of the treated cancer cells to re-grow and re-form colonies after Solamargine treatment (Fig. 1d, e). The number of colonies produced by WM239 cells after treatment with the IC_50_ of Solamargine was significantly reduced. The control (DMSO) treated cells WM35, WM115, and WM239 cells produced an average of 71.3 ± 2.9, 85.0 ± 4.6, and 97.3 ± 1.0 colonies/10^4^ cells, respectively. However after IC_50_ Solamargine treatment for 2 h, there was significant reduction in the number of colonies subsequently formed by the three cell lines with a maximum effect on the metastatic cell line WM239 which formed an average of only 8.7 ± 1.4 colonies/10^4^ cells (*P* < 0.05) (Fig. 1d). Consistent with the results of the short-term toxicity assays, RGP WM35 cells displayed minor reductions in clonogenic survival upon Solamargine treatment.

Similarly, the number of colonies produced by the WM115 and WM239 cells was significantly reduced after treatment with the IC_70_ of Solamargine for 2 h (Fig. 1e). The control (DMSO) group of WM35, WM115, and WM239 cells produced an average of 71.3 ± 2.9, 93.0 ± 0.5, and 107.3 ± 3.7 colonies/10^4^ cells, respectively, while the treated groups exhibited 63.0 ± 2.9 (*P* < 0.05), 16.0 ± 0.8 (*P* < 0.05), and 3.0 ± 0.5 (*P* < 0.05) colonies/10^4^ cells, respectively (Fig. 1e).

### Cellular morphology changes upon Solamargine treatment

**Fig. 2:**
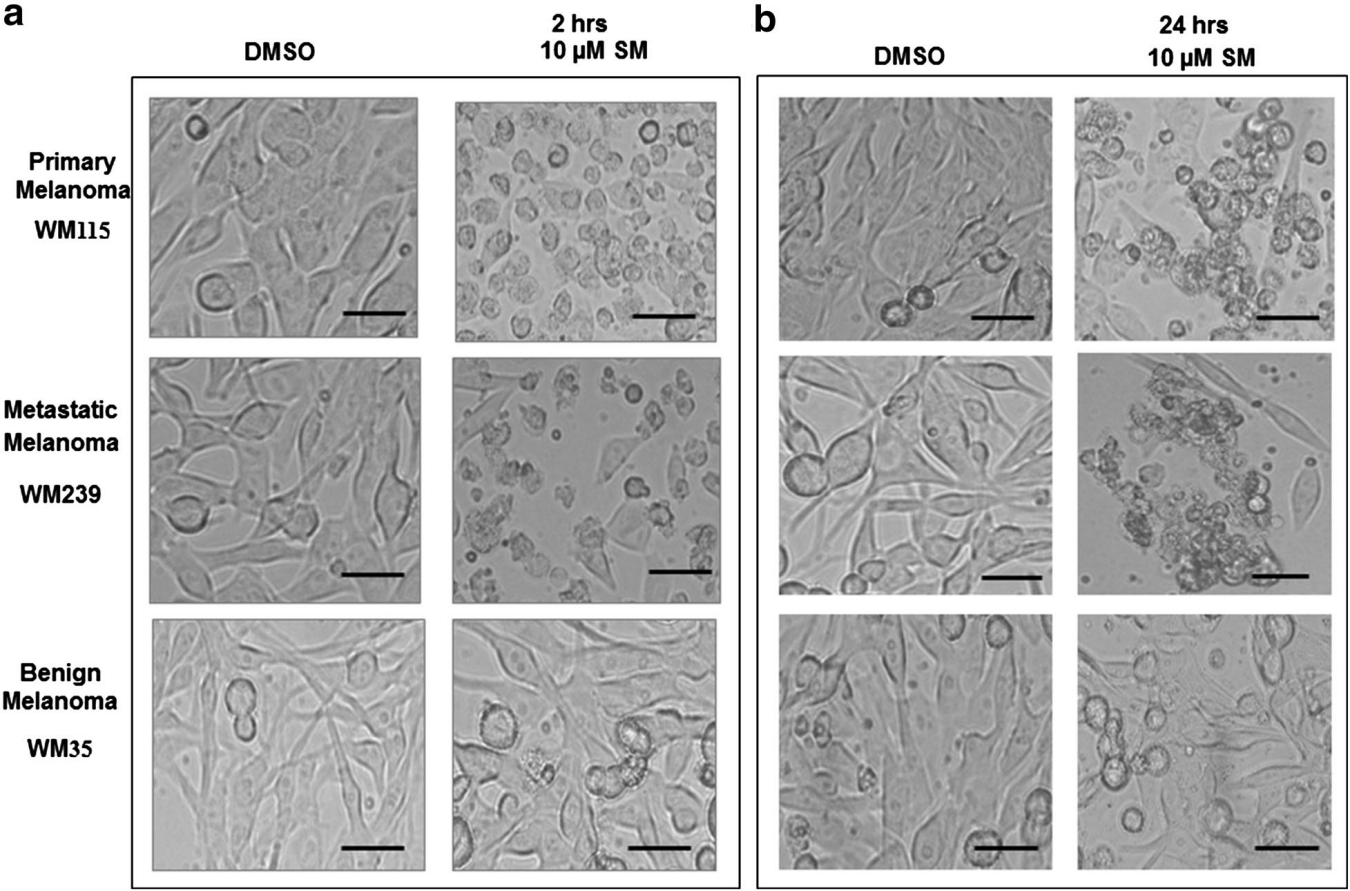
Light microscope images shows that 10 µM Solamargine deformed the malignant but not benign cells. **a** 2 and **b** 24 h Solamargine induced morphological changes such as cellular rounding up, shrinkage and detachment in malignant WM115 and WM239 cells, but no such changes were observed in benign WM35 melanoma cells. *Scale bar* 50 µm

The cells were monitored microscopically after the first 2 h of the drug administration and followed up to 24 h along with control treatment (DMSO). As shown in Fig. 2, WM115 and WM239 cells initially became rounded in shape and smaller in size, and subsequently lost contact with neighboring cells and floated in the medium. The morphology of WM35 cells was not changed even after 24 h of 10 µM of Solamargine as compared to its respective control (Fig. 2).

### Flow cytometry analysis of cellular death events

**Fig. 3:**
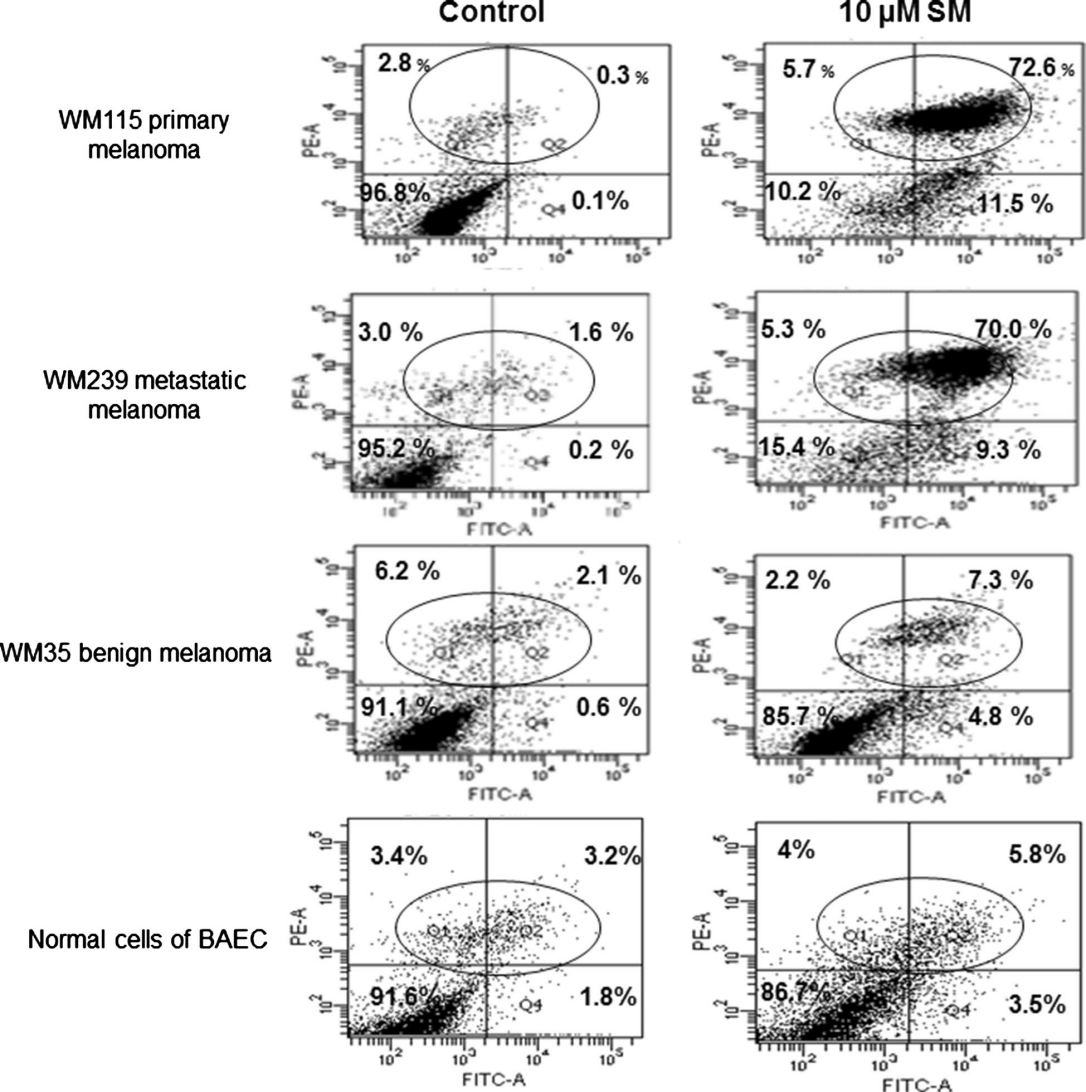
Flow cytometry analysis showed, Solamargine increased apoptosis tenfold more in malignant to benign cells. 10 µM Solamargine treatment for 2 h selectively caused massive cellular death of WM115 primary and WM239 metastatic melanoma cells which was at least tenfold more than the detected in benign WM35 and/or in BAEC cells.The results shown are for one representative experiment of triplicate determinations

Since the decrease in cellular proliferation may be a result of the induction of apoptosis or necrosis, we quantified the number of apoptotic versus necrotic cells in both Solamargine treated and control cells at different time points. As shown in Fig. 3, treatment of melanoma cells with Solamargine (10 µM) rapidly induced late apoptosis and necrosis as demonstrated by the rapid increase in the percentage of the apoptotic (annexin V-positive) and necrotic (PI-positive) cells. Most of the PI-stained necrotic cells were detected within the first 2 h of Solamargine treatment. The necrotic effect of Solamargine was less on the WM35 and BAEC cell lines. After treatment with 10 µM Solamargine there was a 1.2 fold increase in the PI-stained (necrotic) WM35 cells compared to control cells and 2.8 fold increase in necrotic BAEC cells, whereas, 16- and 12-fold increases in the PI-stained necrotic cells were seen in WM115 and WM239 cells, respectively. The fold increase of necrosis was calculated as the mean value of four repeated independent flow cytometer experiments; Fig. 3 shows representative data from one of the four flow cytometer runs. The same results were obtained upon exposure to IC_50_ Solamargine treatment (data not shown). These experiments were repeated in four independent runs.

### Transmission electron microscopy analysis revealed that Solamargine caused necrosis in the vertical growth melanoma cell lines selectively

**Fig. 4:**
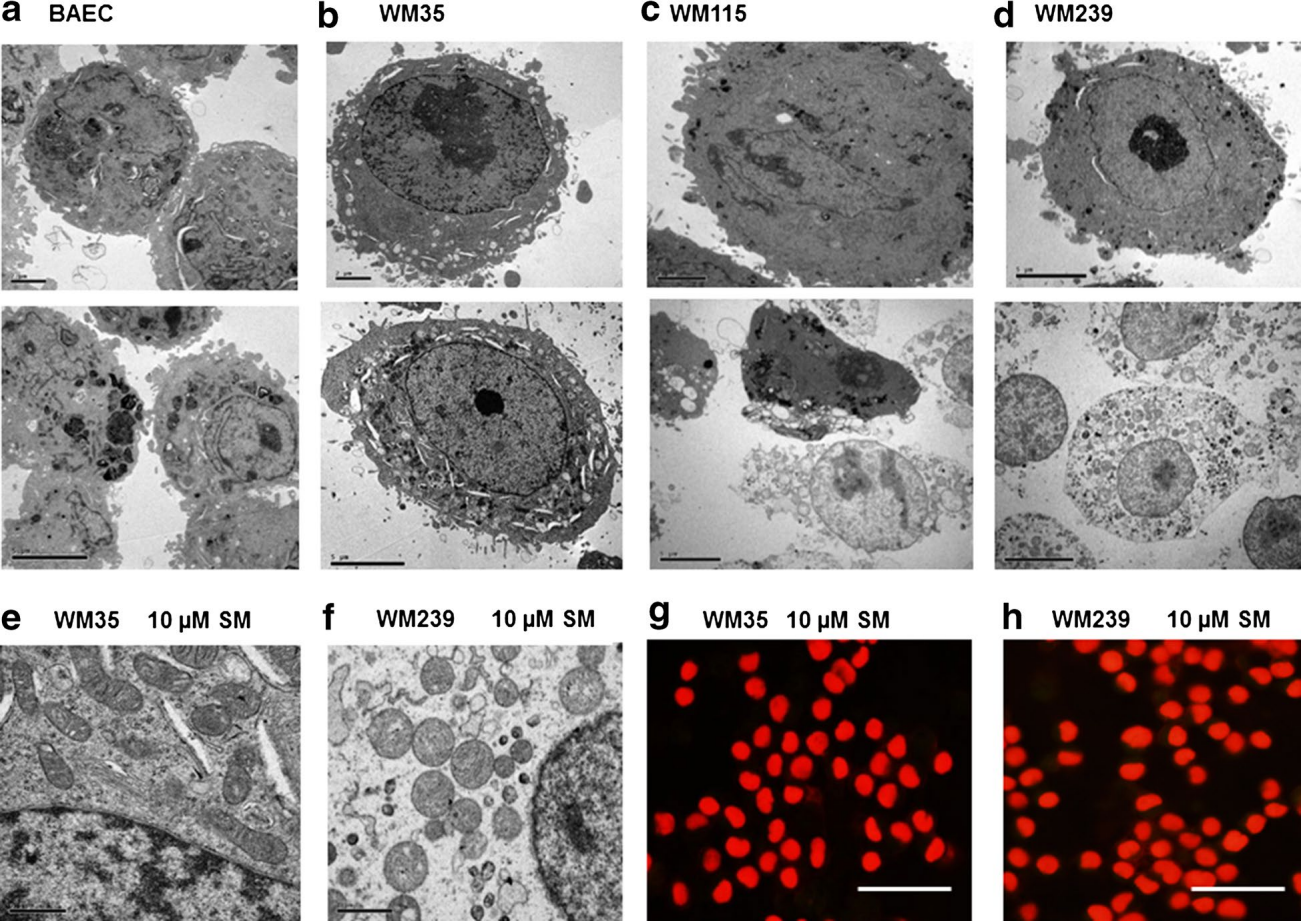
TEM images for BAEC, WM35, WM115 and WM239, shows the effect of Solamargine on cellular ultrastructure and fluorescent images shows intact nuclei after Solamargine treatment in WM35 and WM239 cells. *Top images* show control and the *bottom panels* show 10 µM Solamargine treatment for 2 h. Solamargine induced signs of necrosis in WM115 primary melanoma (**c**) and WM239 metastatic melanoma (**d**) cells, but benign melanoma WM35 (**b**) and BAEC (**a**) cells showed milder cell disruption, *scale bars* 5 µm. **e** and **f** Two higher magnification images show the swollen mitochondria in WM239 cells compared to the normal looking mitochondria of WM35 cells after their exposure to 10 µm of Solamargine for 2 h. *Scale bars* 0.5 µm. **g** and **h** Staining of cultured WM35 and WM239 cells with propidium iodide after 2 h Solamargine treatment shows intact nuclei and no signs of nuclear fragmentation or condensation. *Scale bars* 50 µm

The sub cellular changes associated with Solamargine-induced cell death in melanoma cell lines WM35, WM115, and WM239 and BAEC cells were detected using Transmission Electron Microscopy. The control treatments (DMSO) of the four cell lines BAEC, WM35, WM115, WM239 (Fig. 4 a–d top panel) showed no morphological signs of apoptosis or necrosis, displaying intact cells with uniform rounded shape, very few vesicles (or vacuoles) in the cytoplasm and normal cell organelles. Treatment of these cells with 10 µM Solamargine for 2 h caused morphological signs of necrosis only in WM115 and WM239 cells, characterized as evidence of cell lysis such as disruptions in plasma membrane, swollen mitochondria (Fig. 4f), increased vacuoles/lysosomes (Fig. 4c bottom photo). Some cells showed cytoplasmic vacuolation, increased cell volume, culminating in the disruption of the plasma membrane and cell rupture (green arrow) leading to the leakage of cellular contents in some cells which are morphologic hallmarks of necrosis [23]. The nuclear membrane was intact in all the treated cells, and staining of treated cultured cells with propidium iodide revealed homogeneous nucleic acid distribution with no evidence of nuclear condensation or apoptotic body formation (Fig. 4g, h).

### Solamargine treatment caused upregulation of tumor necrosis factor receptor-1, cytochrome C and cathepsin B and other cell death related proteins in melanoma cells

**Fig. 5:**
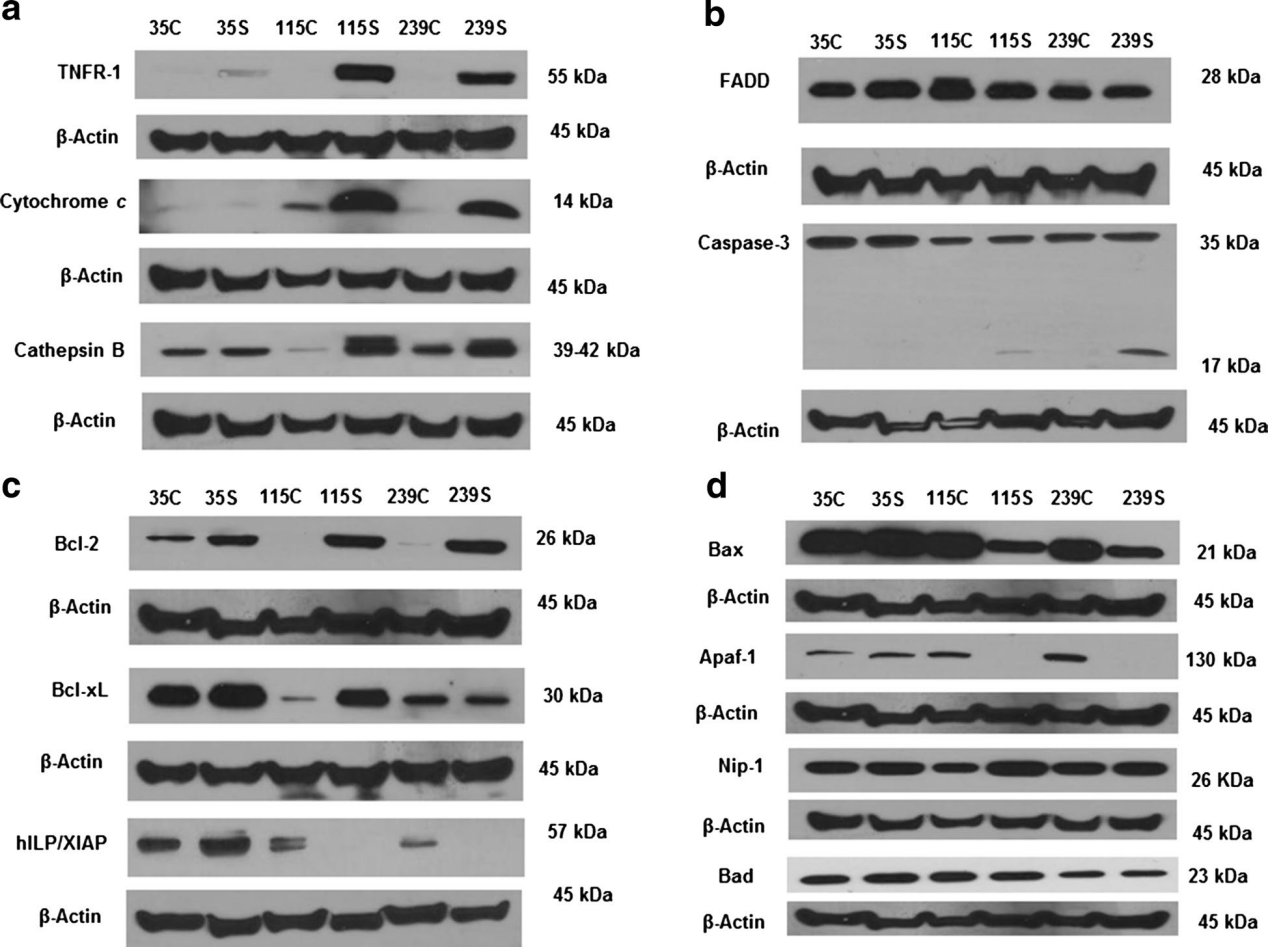
Representative western blots for the cellular death proteins in the presence and absence of Solamargine. **a** TNFR1, cytochrome c, and cathepsin B were up-regulated in malignant WM115 and WM239 cells while these proteins were not significantly altered in the benign WM35 cells. **b** There were no significant changes for FADD protein however, a slight increase in the cleaved caspase-3 protein was observed in both WM115 and WM239 cells, respectively. **c** The anti-apoptotic protein Bcl-2 was up-regulated more profoundly in WM115 and WM239 cells when compared to WM35 cells. Solamargine also increased the expression of Bcl-xL in both WM35 and WM115 cells, with no effect on WM239 cells. The expression of hILP/XIAP was down regulated in the presence of Solamargine only in WM115 and WM239 cells. **d** The pro-apoptotic proteins Bax and Apaf-1 were also down regulated upon the administration of Solamargine in WM115 and WM239, but not WM35 cells. Nip-1 and Bad levels did not change significantly with treatment in any cell lines

**Fig. 6:**
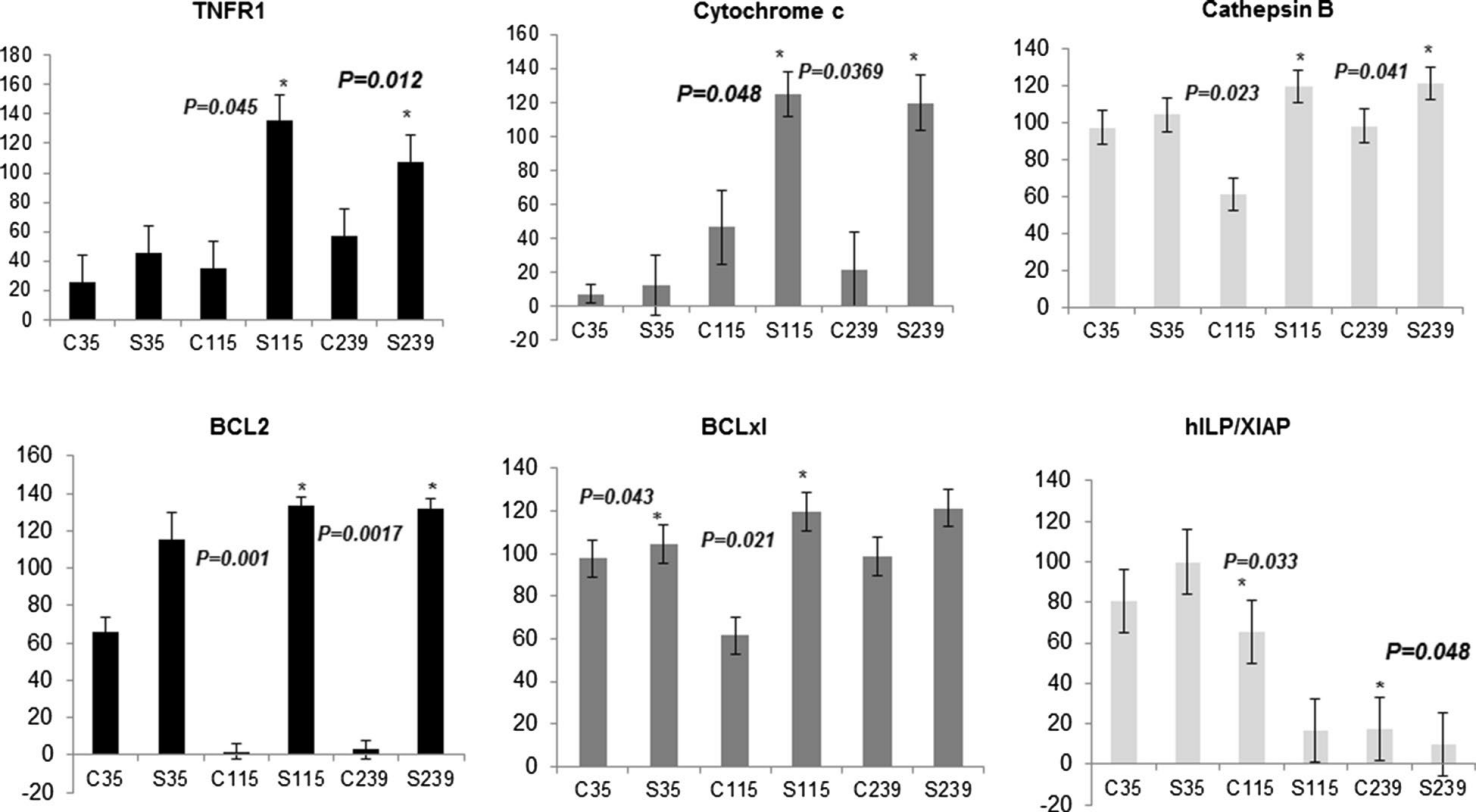
Western blot densitometry and relative intensity of protein bands calculated using image J software. Treatment with Solamargine (10 µM) for 2 h resulted in a significant increase in TNFR1 expression in both WM115 and WM239 cells the when compared with control (DMSO) treated cells (*p* = *0.045* and *p* = *0.012* respectively). Cytochrome c was upregulated significantly after the treatment in both WM115 and WM239 (*p* = *0.048* and *p* = *0.0329* respectively). Cathepsin B had the same pattern as the former proteins and was upregulated upon Solamargine treatment (*p* = *0.023* and *p* = *0.041* respectively. BCL2 protein was also upregulated significantly upon the treatment in both WM115 and WM239 (*p* = *0.0001* and *p* = *0.0017* respectively). Unlike the former proteins Bcl-xL was upregulated significantly in the benign WM35 cells and the primary cells VGP WM115 cells (*p* = *0.043* and *p* = *0.021* respectively). hILP/XIAP was down regulated significantly with the treatment in both malignant cell lines WM115 and WM239 (*p* = *0.033* and *p* = *0.048 respectively*), N = 3

As shown in Figs. 5a and 6, the expression of TNFR-1 was extensively up-regulated in WM115 and WM239 cells treated with 10 μM Solamargine for 2 h compared to control, whereas it was only slightly increased in WM35 cells. Treatment with IC_50_ Solamargine caused the same effect (data not shown). Solamargine (10 µM) also significantly increased the levels of cathepsin B and cytochrome c in WM115 and WM239 cells but not in WM35 cells (Figs. 5a, 6). There was an appreciable increase in the proteolytic activity of caspase-3 resulting in its cleavage in WM239 cells, which was greater than that found in WM115 cells. However this activation of caspase-3 was not detected in WM35 cells. FADD levels were not affected significantly by Solamargine treatment in any melanoma cell line (Fig. 5b).

The anti-apoptotic protein Bcl-2 was up-regulated upon treatment in both WM115 and WM239 and to a less extent in WM35 cells. Furthermore, 10 µM of Solamargine up-regulated the expression of the anti-apoptotic protein Bcl-xL in WM115 and WM35 cells, while no obvious change was observed in WM239 cells (Figs. 5c, 6). The anti-apoptotic protein XIAP was down regulated in WM115 and WM239 cells and was slightly increased in WM35 cells treated with 10 µM Solamargine (Figs. 5c, 6).

The pro-apoptotic proteins Bax, Apaf-1, Nip-1 and Bad were also evaluated by western blot (Fig. 5d). Solamargine (10 µM) treatment for 2 h reduced the expression of Bax and Apaf-1 in WM115 and WM239 cells, whereas the expression of these proteins was not affected in WM35 cells. The expression of Bad and Nip-1 was not affected by 10 µM Solamargine treatment in all three cell lines (Fig. 5d).

## Discussion

Drug discovery and the search for potent and selective anti-cancer agents are valid aims for many scientists worldwide. Early in 1987 Cham et al. showed that extracts from *Solanum* plant species possessed antitumor properties against sarcoma 180 in mice [24]. Later in 2007 they reported the effectiveness of a cream containing Solasodine Rhamnosyl Glycosides (SRGs) in the treatment of large number of non-melanoma skin cancers in humans and horses [11]. In this study we measured the half inhibitory concentrations (IC_50_) of Solamargine on three melanoma cell lines representing benign and malignant forms of the disease. Our results clearly show that Solamargine selectively inhibits the highly proliferating/malignant melanoma cells with minimum necrotic effect on benign melanoma and normal cells. This result is supported by many previous studies done on different types of cancer cells. For instance, Solamargine caused cellular death for breast cancer cells HBL-100, SK-BR-3 and ZR-75-1 after 3 h of their exposure to IC_50_ concentrations of 2.07, 3.00 and 2.15 µM respectively [9]. However, Solamargine at higher concentration (IC_50_, 8.5 µM) was needed to kill human K562 leukaemia cells within 2 h [16]. Human lung cancer cells H441, H520, H661 and H69 displayed positive annexin V staining after 2 h of various concentrations of Solamargine (3–5.8 µM) [25]. All these studies together with the current study indicate that Solamargine has an anti-proliferative effect against cancer cells and the growth inhibitory concentration is highly dependent on the type of cancer cell. To our knowledge this is the first study showing the parallel effect of Solamargine on both normal cells as well as the melanoma cells from early and advanced lesions (RGP and VGP). We found that the colony formation ability of both WM115 and WM239 cells pre-treated with Solamargine was extensively reduced while the benign WM35 cells retained their ability to form a similar number of colonies to control even after their exposure to IC_70_ of the drug. This clonogenic survival test was not performed in the previous studies, although it is considered the gold standard in vitro assay to detect the efficacy of chemotherapy drugs [26].

Flow cytometry analysis indicated that Solamargine caused cellular necrosis in the highly proliferating melanoma cells (WM115 and WM239) upon the treatment with Solamargine at concentrations that did not exert effects on either WM35 cells or BAEC. These results were consistent with caspase-3 analysis that showed relatively low cleavage in both WM115 and WM239 cells. The prominence of the necrosis pathway is further supported by the changes in mitochondrial function. We found at most a 40 % reduction in mitochondrial membrane potential (in WM239 cells) indicating that apoptosis is not the primary cause of the rapid cellular death observed in the first two hours of treatment. Similar flow cytometry results were obtained in different human lung cancer cells (H441, H520, H661, H69) treated with Solamargine which rapidly (in the first hour of incubation) induced apoptosis and increased percentage of annexin V-positive cells but only few double-positive necrotic cells were detected [25].

Solamargine triggered TNFR1 upregulation in the highly proliferative melanoma cells (WM115 and WM239) but TNFR1 was only slightly up regulated in the slow proliferating WM35 cells. This suggests that TNFR1 may mediate Solamargine induced necrosis in those cells, which sensitizes them to lysosomal cathepsin B mediated by the release of mitochondrial cytochrome c. Such effect of cathepsin B was reported in TNFα mediated hepatocyte apoptosis [27]. Consistent with our finding, Sun et al. [16] reported that 7.5 µM of Solamargine could induce early lysosomal rupture in human K562 leukemia cells within 2 h which was confirmed by the release of cathepsin B to cytosol. Previous studies also reported that Solamargine at different concentrations increases the expression of TNFR-1 in various cancer cells, such as lung [25], hepatoma [28], breast [10], and human squamous cell carcinoma [15].

In addition to rapid induction of cell necrosis, we obtained evidence that the intrinsic apoptosis pathway was also affected and altered in WM115 and WM239 cells, perhaps due to unbalance between the pro- and anti-apoptotic proteins. The anti-apoptotic protein XIAP was down regulated in both WM115 and WM239 cells treated with Solamargine, however it was slightly upregulated in WM35 cells. When XIAP is bound to caspase-3, the protease is inhibited, thus if the cell receives a signal to undergo apoptosis, Smac/Diablo proteins released from the mitochondria bind to XIAP freeing up caspase-3 for activation and subsequent apoptosis [29]. The above phenomenon may explain our results that both WM115 and WM239 cells loose XIAP expression and display active (cleaved) caspase 3 when treated with Solamargine. However benign radial growth phase WM35 cells express high levels of XIAP even when exposed to Solamargine, and caspase-3 was not cleaved. These results were confirmed by the TEM images which showed clearly that only the WM115 and WM239 cells had evidence of loss of plasma membrane integrity and cell rupture. The nuclei in the treated WM115 and WM239 cells looked normal which indicates that these morphological changes are due to necrotic and not apoptotic effects of the drug, since necrotic cells do not fragment into discrete corpses as their apoptotic counterparts do. Moreover, their nuclei remain intact and can aggregate and accumulate in necrotic tissues [30].

Solamargine treatment up-regulated Bcl2 expression in these cells, suggestive of a protective effect. It was previously reported that Bcl2 inhibition sensitized resistant melanoma cells to Apo2/TRAILl (TNF related apoptosis-inducing ligand) [31]. The related prosurvival protein Bcl-xL was highly expressed in control radial growth phase WM35 cells and levels slightly increased upon treatment. Bcl-xL profoundly increased in WM115 upon Solamargine exposure however, its level was not changed in WM239 cells. Previous reports showed that the expression of Bcl-xL protein is responsible for melanoma cell chemoresistance, and inhibiting this anti-apoptotic protein increased the chemosensitivity of the cells [7]. Therefore the increase in these two anti-apoptotic proteins seen in our studied cells might be as a controversial response to counter the action of the drug.

## Conclusions

Taken together our results suggest that the mitochondrial pathway is involved in Solamargine-induced cell death in human malignant melanoma cells [32]. The disruption of the mitochondrial membrane in Solamargine treated cells was confirmed by electron microscopy. However the classic mitochondrial apoptosis pathway (intrinsic pathway) is apparently not involved in the Solamargine-induced cell death both decreases in pro-apoptotic protein expression and increases in anti-apoptotic protein expression in the studied cells.

Cancer cells are growing and dividing much faster than normal cells, thus molecules which can potently act to quickly stop their proliferation is of high demand for any oncology therapeutic setting. Solamargine shows potential features as a promising future drug since it acted selectively to rapidly cause necrosis to the highly proliferating VGP melanoma cells, but had minimum necrotic effect on the benign RGP melanoma and normal cells. Since most melanomas show clinical resistance to anti-apoptotic drugs [5], Solamargine holds promise as a necrotic inducer for the more serious malignant stage melanoma.

## Additional file

10.1186/s12935-016-0287-4 Antibodies used and their dilutions, Isotype, catalogue number and source.

## Abbreviations

VGP: vertical growth phase
RGP: radial growth phase
TEM: transmission Electron Microscope
TNFR1: tumor necrosis factor receptor 1
hILP: human IAP-like Protein
XIAP: X-linked inhibitor of apoptosis protein
Bcl-xL: B-cell lymphoma-extra-large
Apaf-1: apoptotic protease activating factor 1
Bax: Bcl-2-associated X protein

## References

[1] Leiter U, Eigentler T, Garbe C (2014). Epidemiology of skin cancer. Adv Exp Med Biol.

[2] Geller AC, Clapp RW, Sober AJ, Gonsalves L, Mueller L, Christiansen CL (2013). Melanoma epidemic: an analysis of six decades of data from the connecticut tumor registry. J Clin Oncol.

[3] Siegel R, Ma J, Zou Z, Jemal A (2014). Cancer statistics, 2014. CA Cancer J Clin.

[4] Czarnecki D (2014). The incidence of melanoma is increasing in the susceptible young Australian population. Acta Derm Venereol.

[5] Wouters J, Stas M, Gremeaux L, Govaere O, Van den Broeck A, Maes H (2013). The human melanoma side population displays molecular and functional characteristics of enriched chemoresistance and tumorigenesis. PLoS One.

[6] Serrone L, Hersey P (1999). The chemoresistance of human malignant melanoma: an update. Melanoma Res.

[7] Heere-Ress E, Thallinger C, Lucas T, Schlagbauer-Wadl H, Wacheck V, Monia BP (2002). Bcl-x(L) is a chemoresistance factor in human melanoma cells that can be inhibited by antisense therapy. Int J Cancer.

[8] Viudez A, Ramirez N, Hernandez-Garcia I, Carvalho FL, Vera R, Hidalgo M (2014). Nab-paclitaxel: a flattering facelift. Crit Rev Oncol Hematol.

[9] Shiu L, Chang L, Liang C, Huang Y, Sheu H, Kuo K (2007). Solamargine induces apoptosis and sensitizes breast cancer cells to cisplatin. Food Chem Toxicol.

[10] Shiu L-Y, Liang C-H, Huang Y-S, Sheu H-M, Kuo K-W (2008). Downregulation of HER2/neu receptor by solamargine enhances anticancer drug-mediated cytotoxicity in breast cancer cells with high-expressing HER2/neu. Cell Biol Toxicol.

[11] Cham BE (2007). Solasodine rhamnosyl glycosides in a cream formulation is effective for treating large and troublesome skin cancers. Res J Biol Sci.

[12] Friedman M, McDonald GM, Filadelfi-Keszi M (1997). Potato glycoalkaloids: chemistry, analysis, safety, and plant physiology. Crit Rev Plant Sci.

[13] Ma-C Sánchez-Mata (2010). Yokoyama WE, Hong Y-J, Prohens J. α-Solasonine and α-solamargine contents of gboma (*Solanum macrocarpon* l.) and scarlet (*Solanum aethiopicum* l.) eggplants. J Agric Food Chem.

[14] Fulda S, Debatin KM (2006). Extrinsic versus intrinsic apoptosis pathways in anticancer chemotherapy. Oncogene.

[15] Wu C-H, Liang C-H, Shiu L-Y, Chang L-C, Lin T-S, Lan C-CE (2011). *Solanum incanum* extract (SR-T100) induces human cutaneous squamous cell carcinoma apoptosis through modulating tumor necrosis factor receptor signaling pathway. J Dermatol Sci.

[16] Sun L, Zhao Y, Li X, Yuan H, Cheng A, Lou H (2010). A lysosomal–mitochondrial death pathway is induced by solamargine in human K562 leukemia cells. Toxicol In Vitro.

[17] Cornil I, Theodorescu D, Man S, Herlyn M, Jambrosic J, Kerbel R (1991). Fibroblast cell interactions with human melanoma cells affect tumor cell growth as a function of tumor progression. Proc Natl Acad Sci.

[18] Bani MR, Rak J, Adachi D, Wiltshire R, Trent JM, Kerbel RS (1996). Multiple features of advanced melanoma recapitulated in tumorigenic variants of early stage (radial growth phase) human melanoma cell lines: evidence for a dominant phenotype. Cancer Res.

[19] Rodeck U, Herlyn M, Menssen HD, Furlanetto RW, Koprowsk H (1987). Metastatic but not primary melanoma cell lines grow in vitro independently of exogenous growth factors. Int J Cancer.

[20] McDiarmid HM, Douglas GR, Coomber BL, Josephy PD (2001). Epithelial and fibroblast cell lines cultured from the transgenic BigBlue rat: an in vitro mutagenesis assay. Mutat Res.

[21] Sher I, Adham SA, Petrik J, Coomber BL (2009). Autocrine VEGF-A/KDR loop protects epithelial ovarian carcinoma cells from anoikis. Int J Cancer.

[22] Adham SA, Sher I, Coomber BL (2010). Molecular blockade of VEGFR2 in human epithelial ovarian carcinoma cells. Lab Invest.

[23] Kressel M, Groscurth P (1994). Distinction of apoptotic and necrotic cell death by in situ labelling of fragmented DNA. Cell Tissue Res.

[24] Cham BE, Gilliver M, Wilson L (1987). Antitumour effects of glycoalkaloids isolated from Solanum sodomaeum. Planta Med.

[25] Liu L-F, Liang C-H, Shiu L-Y, Lin W-L, Lin C-C, Kuo K-W (2004). Action of solamargine on human lung cancer cells–enhancement of the susceptibility of cancer cells to TNFs. FEBS Lett.

[26] Munshi A, Hobbs M, Meyn RE (2005). Clonogenic cell survival assay. Methods Mol Med.

[27] Guicciardi ME, Deussing J, Miyoshi H, Bronk SF, Svingen PA, Peters C (2000). Cathepsin B contributes to TNF-alpha-mediated hepatocyte apoptosis by promoting mitochondrial release of cytochrome c. J Clin Invest.

[28] Kuo K-W, Hsu S-H, Li Y-P, Lin W-L, Liu L-F, Chang L-C (2000). Anticancer activity evaluation of the solanum glycoalkaloid solamargine: triggering apoptosis in human hepatoma cells. Biochem Pharmacol.

[29] Wang K, Lin B (2013). Inhibitor of apoptosis proteins (IAPs) as regulatory factors of hepatic apoptosis. Cell Signal.

[30] Vandenabeele P, Galluzzi L, Berghe TV, Kroemer G (2010). Molecular mechanisms of necroptosis: an ordered cellular explosion. Nat Rev Mol Cell Biol.

[31] Chawla-Sarkar M, Bae SI, Reu FJ, Jacobs BS, Lindner DJ, Borden EC (2004). Downregulation of Bcl-2, FLIP or IAPs (XIAP and survivin) by siRNAs sensitizes resistant melanoma cells to Apo2L/TRAIL-induced apoptosis. Cell Death Differ.

[32] Lewis JS, Meeke K, Osipo C, Ross EA, Kidawi N, Li T (2005). Intrinsic mechanism of estradiol-induced apoptosis in breast cancer cells resistant to estrogen deprivation. J Natl Cancer Inst.

