# Supplementary material for: Solamargine triggers cellular necrosis selectively in different types of human melanoma cancer cells through extrinsic lysosomal mitochondrial death pathway
Source: Cancer Cell Int. 2016 Feb 17;16:11. doi: 10.1186/s12935-016-0287-4 (PMC4756414; doi:10.1186/s12935-016-0287-4)
Supplement: Supplementary file 1 — 10.1186/s12935-016-0287-4 Antibodies used and their dilutions, Isotype, catalogue number and source. [file 12935_2016_287_MOESM1_ESM.doc]

Table SI: Antibodies used and their dilutions, Isotype, catalogue number and source.

| Antibody | Isotype | Dilution | Company | Cat # |
| --- | --- | --- | --- | --- |
| Apaf-1 | Mouse antibody | 1:500 | BD Transduction Laboratories | Apoptosis I Sampler Kit# 612741 |
| Bad |  | 1:250 |  |  |
| Bax |  | 1:500 |  |  |
| Bcl-2 |  | 1:250 |  |  |
| Bcl-xl |  | 1:250 |  |  |
| hILP/XIAP |  | 1:500 |  |  |
| Nip1 |  | 1:500 |  |  |
| Cathepsin B | Rabbit antibody | 1:1000 | Cell Signaling Technology | 3373S |
| TNF-R1 |  | 1:1000 |  | 3736S |
| Cytochrome c |  | 1:1000 |  | 4272S |
| Caspase 3 |  | 1:1000 |  | 9654S |
| FADD |  | 1:1000 |  | 2782S |
